# Comparison of a dichotomous versus trichotomous checklist for neonatal intubation

**DOI:** 10.1186/s12909-022-03700-4

**Published:** 2022-08-26

**Authors:** Lindsay Johnston, Taylor Sawyer, Akira Nishisaki, Travis Whitfill, Anne Ades, Heather French, Kristen Glass, Rita Dadiz, Christie Bruno, Orly Levit, Marc Auerbach

**Affiliations:** 1 Department of Pediatrics, Yale University School of Medicine, 333 Cedar Street, New Haven, CT 06510 USA; 2 Department of Pediatrics, University of Washington School of Medicine, Seattle, USA; 3 Department of Anesthesiology and Critical Care Medicine, University of Pennsylvania Perelman School of Medicine, Philadelphia, USA; 4 Department of Pediatrics, University of Pennsylvania Perelman School of Medicine, Philadelphia, USA; 5 Department of Pediatrics, Penn State College of Medicine, Hershey, USA; 6 School of Medicine and Dentistry, Department of Pediatrics, University of Rochester, Rochester, USA

**Keywords:** Neonatal intubation, Dichotomous checklist, Trichotomous checklist, Global skills assessment, Entrustable professional activities assessment

## Abstract

**Background:**

To compare validity evidence for dichotomous and trichotomous versions of a neonatal intubation (NI) procedural skills checklist.

**Methods:**

NI skills checklists were developed utilizing an existing framework. Experts were trained on scoring using dichotomous and trichotomous checklists, and rated recordings of 23 providers performing simulated NI. Videolaryngoscope recordings of glottic exposure were evaluated using Cormack-Lehane (CL) and Percent of Glottic Opening scales. Internal consistency and reliability of both checklists were analyzed, and correlations between checklist scores, airway visualization, entrustable professional activities (EPA), and global skills assessment (GSA) were calculated.

**Results:**

During rater training, raters gave significantly higher scores on better provider performance in standardized videos (both *p* < 0.001). When utilized to evaluate study participants’ simulated NI attempts, both dichotomous and trichotomous checklist scores demonstrated very good internal consistency (Cronbach’s alpha 0.868 and 0.840, respectively). Inter-rater reliability was higher for dichotomous than trichotomous checklists [Fleiss kappa of 0.642 and 0.576, respectively (*p* < 0.001)]. Sum checklist scores were significantly different among providers in different disciplines (*p* < 0.001, dichotomous and trichotomous). Sum dichotomous checklist scores correlated more strongly than trichotomous scores with GSA and CL grades. Sum dichotomous and trichotomous checklist scores correlated similarly well with EPA.

**Conclusions:**

Neither dichotomous or trichotomous checklist was superior in discriminating provider NI skill when compared to GSA, EPA, or airway visualization assessment. Sum scores from dichotomous checklists may provide sufficient information to assess procedural competence, but trichotomous checklists may permit more granular feedback to learners and educators. The checklist selected may vary with assessment needs.

**Supplementary Information:**

The online version contains supplementary material available at 10.1186/s12909-022-03700-4.

## Background

Given concerns for limited clinical procedural exposure for trainees [1, 2], medical educators must identify effective strategies to ensure procedural proficiency. Simulation is frequently utilized for skills training with numerous authors reporting improved patient outcomes after rigorous simulation-based procedural training [3–5]. A key requirement for simulation-based procedural training is an effective method to measure performance compared to a defined standard. Observational assessment tools are utilized to measure provider performance. Evaluation of multiple sources of validity evidence [6–9] (such as content, response process, internal structure, relations to other variables, and consequences [9]) is critical when developing or selecting an assessment tool.

An important component of a validity argument is ensuring that the selected tool actually measures the construct of interest, enabling an educator to have confidence in making learner assessments based upon the results obtained [10]. Numerous types of observational assessment tools exist, including global skills assessments (GSA), entrustable professional activities (EPA) assessments, and procedural skills checklists (Table 1). The GSAs provide a rating of an entire procedural encounter [11], whereas EPAs require supervisors to judge the level of autonomy a trainee should be permitted clinically [12, 13]. The GSAs and EPAs are based upon the rater’s general impression of the learner’s performance utilizing an anchored rating scale, while checklists present discrete observable actions to objectively rate performance 14]. Checklists may be utilized for formative assessment, when immediate, granular feedback is provided to improve performance, or summative assessment, when official judgments about performance (eg, credentialing or training advancement) are required. An observational assessment tool’s value is largely dependent on the rigor dedicated to the tool’s creation, and the rater’s experience, training, and calibration in using the tool [8]. The importance of these factors increases substantially with high-stakes assessments [15].

**Table 1:** Differentiation of Various Types of Observational Assessment Tools

Type of Tool	Subtype (if applicable)	Description	Rating Scale Utilized	Potential Uses, Benefits, and Challenges
**Procedural Skills Checklists **[14]	Dichotomous	Utilizes discrete observable actions to objectively rate performance	Binary options (yes/ no) for rating whether each specific action was performed using correct technique	• Useful in procedural preparedness • Ensures all actions are performed using appropriate sequence and technique • Limited subjectivity, as ratings are based upon observable actions • Valuable for summative assessment
	Trichotomous	Utilizes discrete observable actions to objectively rate performance	Three options for rating whether each specific action was performed 1) correctly (full credit), 2) required alteration in technique (partial credit), or 3) not performed/ performed incorrectly (no credit)	• Useful in procedural preparedness • Ensures all actions are performed using appropriate sequence and technique • Limited subjectivity, as ratings are based upon observable actions • Additional rating options present opportunity for formative feedback
**Global Skills Assessment (GSA) **[11]	N/A	Utilizes rater’s general impression of learner’s procedural performance	Behaviorally-anchored rating scale (ex., ranging from “novice” to “expert” performance)	• Assessment of entire procedural skill (clinical or simulated) • Less granular feedback than with checklist • Often valuable in assessing expert performance
**Entrustable Professional Activities Assessment (EPA) **[12, 13]	N/A	Utilizes rater’s general impression of learner’s performance to judge the level of clinical autonomy which should be permitted	Anchored rating scale describing degree of entrustment in various clinical procedural situations (ex., ranging from observing only to supervising procedural training of junior learners)	• Assessment of entire procedural skill (clinical or simulated) • No granular feedback unless combined with another tool • Useful in graduate medical education to inform entrustment decisions and to provide summative assessments

Two subtypes of checklists, dichotomous and trichotomous, are addressed in this study. Dichotomous checklists present binary options for rating whether a particular item is completed using the correct technique. Trichotomous checklists broaden each item’s rating options to three choices depending if the correct action is performed using the appropriate technique (full credit), if prompting or alteration in technique was required (partial credit), or if the item was not performed or performed incorrectly (no credit). It is currently unclear whether there are educational benefits associated with the use of dichotomous versus trichotomous checklists for procedural skills training. The current study seeks to address this gap using a checklist designed to assess neonatal endotracheal intubation (NI), a critically important procedure for providers working in the neonatal intensive care unit (NICU). The purpose of this study was to compare dichotomous and trichotomous versions of the NI checklist and correlate each with provider discipline and experience, objective assessment of airway visualization, and subjective performance assessment using EPA and GSA to evaluate for potential differences in discrimination of provider skill level. We hypothesized that, in a simulation-based model of NI performance assessment of pediatric providers at varying skill levels, there would be improved ability to discriminate provider NI skills using dichotomous versus trichotomous checklist scores when compared to other assessment measures, and that dichotomous checklists would have better inter-rater reliability compared to trichotomous versions.

## Methods

### Checklist development

Two versions of a novel observational assessment tool for NI were developed using guidelines developed by the INSPIRE (International Network for Simulation-Based Pediatric Innovation, Research, and Education) Research Network (http://inspiresim.com) [16] as previously described [17]. The observational assessment tools consist of several components:

- Procedural skills checklist: A previously published checklist for nasotracheal NI [18] was adapted for orotracheal intubation using the INSPIRE template. Content was refined through expert consensus and a modified Delphi process with international geographic representation. The 22-item checklist included 6 items addressing procedural preparation, 13 discrete procedural steps, and 3 post-procedural considerations. Two distinct checklist versions were developed.
  ο Dichotomous: Rating choices for each item were binary, with awarded points of 1 (“Done correctly”), 0 (“Done incorrectly; not done”), or N/A (“Not needed; not applicable”). This version was felt to be appropriate for summative assessment, as it focuses on the correctness of each step with less subjectivity.
  ο Trichotomous: Three rating options existed for each item, with awarded points ranging from 2 (“Done independently; Done correctly”), 1 (“Done with prompt; Done partially”), 0 (“Not done; Done incorrectly”), or N/A (“not applicable”). This version was felt to be beneficial for formative assessments, as it provides details on areas for improvement.
- Both dichotomous and trichotomous observational assessment tools were designed to include identical GSA and EPA assessments [19] to permit comparisons and assess relationships between scores on each individual component.
  ο Global skills assessments (GSA): Measures the rater’s overall assessment of procedural performance using a 5-point ordinal scale with behavioral anchors detailing the continuum between “novice” and “expert” procedural performance.
  ο Entrustable professional activities (EPA): Assesses procedural development relevant to the degree of entrustment the rater feels the learner should be given in clinical situations using a 5-point ordinal scale. Providers given the lowest rating are recommended to observe clinical procedures only, while providers on the highest end of scale appear to be qualified to supervise procedural training of junior learners.

The final versions of both assessment tools are available in Appendices A (dichotomous) and B (trichotomous). For convention, “checklist” refers to the skills checklist portion alone (with specificity regarding dichotomous or trichotomous, as applicable), “GSA” or “EPA” as these portions alone, and “observational assessment tool” for either version of the tool in its entirety (checklist + GSA + EPA). Following development, both dichotomous and trichotomous observational assessment tools were evaluated in a simulation-based setting for validity [8, 20]. Correlations were made between dichotomous and trichotomous checklist ratings, GSA and EPA ratings, and objective rating of recorded airway visualization using two previously described measures, the Cormack-Lehane scale [21] [CL; 4-point ordinal scale rating glottic exposure from non-existent (1) to complete (4)] and the Percent of Glottic Opening [22, 23] [POGO; continuous scale rating glottic exposure from non-existent (0%) to complete (100%)], as well as the providers’ discipline and years of experience performing NI.

### Rater training and calibration

Rater training and calibration have been described previously [17], and rater training materials can be found in Appendices C, D, and E. Four board-certified attending neonatologists with > 5 years of experience were recruited from academic centers other than Yale to serve as study raters. They participated in a 2-h training session via teleconference to familiarize themselves with dichotomous and trichotomous observational assessment tools and practice utilizing both versions to rate 3 scripted videos representing expert, competent, and novice NI performance. Following independent rating of each video, a study investigator reviewed the “gold standard” ratings for each item that had been developed by consensus amongst the study team. If the raters scored items differently, there was discussion to ensure that all raters developed a shared understanding of how to utilize the checklists to score various provider actions.

### Data collection

The study protocol was approved by the Human Subjects Committee at Yale University (HSC # 1,505,015,862). Following rater training, a convenience sample of 23 providers [medical students, pediatric residents, neonatology fellows, advanced pediatric providers (APPs), and attending neonatologists] from the academic level IV NICU at Yale-New Haven Children’s Hospital was enrolled after obtaining informed consents between July–August 2015. During a simulation-based scenario, each subject performed a single non-emergent NI attempt on a Laerdal SimNewB mannequin (Laerdal Medical, Stavanger, Germany). The setting and equipment were identical for each provider, and a standardized participant was present to assist. A video camera recorded across the warmer bed, with a view from the manikin’s right side. A Storz C-MAC videolaryngoscope (VL) (Karl Storz Endoskope, Tuttingen, Germany) was used to perform the procedure using direct laryngoscopy, allowing real-time visualization and recording of the inside airway view. Following completion of the study activities, each participant received individualized feedback and coaching to optimize their NI procedural skills. They were encouraged to practice until they were able to successfully intubate the mannequin using proper technique.

External video recordings of each NI attempt were scored by the four trained raters sequentially with dichotomous and trichotomous observational assessment tools. The raters were blinded to the providers’ background information such as training levels.

### Statistical Analysis

#### Rater training and calibration

Cronbach’s alpha and intraclass correlation coefficient (ICC) were calculated based upon internal consistency of rater’s scores for the scripted videos, and agreement of the rater’s calibration to reference scores was assessed using Cohen’s Kappa (*K*). Internal consistency for dichotomous and trichotomous checklists was assessed by calculating Cronbach’s alpha coefficient [24] and item discrimination statistics for all subjects using mean rater scores. Inter-rater reliability (IRR) for airway visualization (CL, POGO) using recorded VL footage was calculated using the kappa statistic and ICC, respectively. The IRR metrics for raters’ assessments with dichotomous and trichotomous checklists were calculated using Fleiss’ kappa statistic for multiple raters [25].

#### Subject performance

Ratings of video recorded simulated NI performance using dichotomous and trichotomous checklists were evaluated in relation to scores on GSA, EPA, and degree of glottic exposure (CL, POGO). The relationship between participant characteristics and dichotomous and trichotomous checklist scores was assessed with one-way ANOVA. Correlation coefficients between continuous variables (i.e., checklist scores) were calculated with the Pearson coefficient; correlation coefficients between a continuous variable and an ordinal variable (i.e., GSA, EPA, CL, and POGO) were calculated with Spearman’s rho coefficient.

Statistical significance was set at a level of *p* < 0.05. Fleiss’ kappa statistics were completed with using R version 3.2.2 (Vienna, Austria). All other analyses were completed with SPSS 22.0 software package (SPSS Inc., Chicago, IL, USA).

## Results

### Rata training data analysis

Four raters completed the training. The internal consistency of rater’s scores in dichotomous and trichotomous checklist scores were assessed (Cronbach’s alpha 0.80–0.92 and 0.76–0.90, respectively). The rater’s initial agreement to reference scores generated by expert consensus was dichotomous: 0.26–0.63 and trichotomous: 0.25–0.49. Their mean dichotomous and trichotomous sum checklist scores for 3 scripted videos representing novice, competent, and expert NI performance were dichotomous (4.9 ± 2.1, 5.6 ± 2.2, 14.3 ± 1.3, *p* < 0.001, one-way ANOVA) and trichotomous (12.1 ± 2.8, 14.7 ± 4.0, 29.4 ± 3.3, *p* < 0.001, one-way ANOVA).

### Dichotomous and trichotomous checklist scores on different levels of providers

Both dichotomous and trichotomous checklist scores demonstrated very good internal consistency (Cronbach’s alpha of 0.868 and 0.840 for dichotomous and trichotomous checklists, respectively). The IRR metrics for raters’ assessments with dichotomous and trichotomous checklists were significantly different; Fleiss kappa for dichotomous checklist scores was 0.642 and for trichotomous scores was 0.576, (*p* < 0.001).

Both dichotomous and trichotomous sum checklist scores effectively discriminated providers with different levels of NI experience during a simulated NI attempt, Table 2. The ranges of the sum checklist scores were 0–18 (dichotomous) and 0–36 (trichotomous), respectively. Sum scores on both dichotomous and trichotomous checklists by students and residents were lower than APPs, fellows, and attendings. Sum checklist scores were significantly different among providers in different disciplines (*p* < 0.001, both dichotomous and trichotomous).

**Table 2:** Dichotomous and trichotomous checklist sum scores (mean, SD) vs. participant role

	Summative score^a^			
	Dichotomous		Trichotomous	
	Mean	*p*-value*	Mean	*p*-value
		< 0.001		< 0.001
Student	2.0 ± 0.0		6.5 ± 0.0	
PA/NNP^b^	12.5 ± 1.8		26.0 ± 3.0	
Residents	6.7 ± 1.6		17.3 ± 2.7	
Fellows	12.8 ± 2.9		26.4 ± 5.5	
Attendings	11.4 ± 3.0		23.6 ± 4.7	

^a^Possible score for dichotomous checklist ranged 0- 18 and highest possible score for trichotomous checklist ranged 0–36. *p-values calculated with one-way ANOVA

^b^PA/NNP refers to physician assistant or neonatal nurse practitioner

Table 3 details mean scores for dichotomous and trichotomous checklists with respect to number of successful clinical NI reported by participants. There was an increase in the sum score with increasing numbers of NI up to 100. However, the most experienced providers (> 100 successful intubations) achieved lower mean scores on both dichotomous and trichotomous checklists when compared to providers with 30–100 intubations. A statistically significant difference was noted between these experience categories for dichotomous and trichotomous checklist scores using a one-way ANOVA (*p* < 0.001 and 0.002, respectively).

**Table 3:** Checklist sum scores vs. number of clinical intubations

	Sum score on Procedural Skills Checklist^a^			
	Dichotomous		Trichotomous	
	Mean	*p*-value*	Mean	*p*-value
Number of successful clinical intubations		< 0.001		0.002
0	6.3 ± 2.3		16.1 ± 5.1	
1–29	10.0 ± 3.2		22.2 ± 5.1	
30–100	13.8 ± 1.6		27.9 ± 2.9	
≥ 100	10.5 ± 2.9		22.4 ± 4.9	

^a^Highest possible score for dichotomous checklist is 18 and highest possible score for trichotomous checklist is 36. *p-values calculated with one-way ANOVA

Table 4 describes correlations between checklist scores and provider characteristics (provider discipline, years of experience, and successful clinical intubations). Both dichotomous and trichotomous sum checklist scores and provider characteristics were not significantly correlated.

**Table 4:** Correlation between checklist variables and provider characteristics

	Provider role	*p*-value	Years experience	*p*-value	Number of intubations	*p*-value
Dichotomous	0.29	0.291	0.41	0.055	0.28	0.197
Trichotomous	0.08	0.730	0.36	0.099	0.22	0.331

Correlation coefficients between two continuous variables were calculated with the Pearson coefficient. Correlation coefficients between a continuous variable and an ordinal variable were calculated with Spearman’s rho coefficient

Table 5 represents correlation coefficients between scores on assessment tools (dichotomous and trichotomous sum checklist scores vs. GSA, EPA, CL, and POGO). The dichotomous and trichotomous sum checklist scores were highly correlated with Pearson’s correlation coefficient of 0.984. Dichotomous and trichotomous sum checklist scores were correlated with airway visualization assessments (CL grade, POGO). The dichotomous sum checklist scores correlated highly with GSA, EPA, POGO, and CL grade. Compared to POGO scores, CL score had higher correlation coefficients with both dichotomous and trichotomous sum checklist scores. All correlation coefficients were statistically significant with *p* < 0.05.

**Table 5:** Correlation of dichotomous and trichotomous procedural skills checklists with each other, Global skills assessments, Entrustable Professional Activities assessments, and airway visualization scores

	Dichotomous	Trichotomous
Dichotomous	1.00	–
Trichotomous	0.98	1.00
GSA	0.85	0.79
EPA	0.87	0.81
POGO	0.59	0.62
CL	0.95	0.81

Correlation coefficients between two continuous variables were calculated with the Pearson coefficient. Correlation coefficients between a continuous variable and an ordinal variable were calculated with Spearman’s rho coefficient. All correlation coefficients are statistically significant with *p* < 0.05

Mean scores and standard deviations for dichotomous and trichotomous checklists are presented by the EPA and GSA assessment scores in Table 6. Dichotomous and trichotomous sum checklist scores increased with higher EPA and GSA scores, *p* < 0.001 for both.

**Table 6:** Relation of Entrustable Professional Activities assessments and Global skills assessments to mean checklist score

	Checklist score			
	Dichotomous^a^	*p*-value	Trichotomous^a^	*p*-value
**EPA Score**^**c**^		< 0.001		< 0.001
1	6.0 ± 2.9		15.3 ± 5.9	
2	9.6 ± 2.9		21.8 ± 5.2	
3	11.6 ± 2.2		24.0 ± 3.5	
4	12.9 ± 2.3		26.0 ± 3.8	
5	14.2 ± 1.9		28.8 ± 3.3	
**GSA Score**^**b**^		< 0.001		< 0.001
1	3.8 ± 1.7		15.8 ± .48	
2	8.5 ± 2.0		18.9 ± 1.6	
3	11.0 ± 1.5		23.9 ± 2.8	
4	12.3 ± 2.2		26.8 ± 2.9	
5	13.9 ± 2.1		28.8 ± 2.8	

^a^Highest possible score for dichotomous checklist is 18 and highest possible score for trichotomous checklist is 36. ^b^GSA scores range from 1 “novice” to 5 “expert.” ^c^EPA scores range from 1 (observe clinical procedures only) to 5 (able to supervise junior trainees performing procedures)

## Discussion

The purpose of this research was to compare two versions of an observational assessment tool containing either dichotomous or trichotomous checklists along with GSA and EPA, and relate scores to other measures to ascertain for the difference in discrimination of procedural performance. In general, we found that both versions were similarly able to discriminate amongst providers at differing levels of experience. The dichotomous checklist had higher agreement (Fleiss kappa) among raters compared to the trichotomous checklist.

### Dichotomous versus trichotomous checklist and usability

When utilized to assess study participants’ recorded simulated NI attempts, both dichotomous and trichotomous checklists demonstrated a high level of consistency and acceptable IRR. Fleiss kappa values were superior for dichotomous as compared to trichotomous checklists [26]. Correlations between the various assessment tools were all similarly high.

In high-stakes assessment, where precise measurements and limited subjectivity are essential, these findings support a significant benefit of dichotomous checklists. If granularity offered by trichotomous checklists is desired, this challenge of lower precision might be overcome with more intensive rater training and calibration. However, even in this study, which included a dedicated 2-h training session with formal calibration, agreement on the training videos was still variable, especially with novice-level performance. This may limit the practicality of utilizing trichotomous checklists in practice, especially for summative assessments.

### Relationship between checklist score and airway visualization

As part of a validity argument, determining how an assessment tool performs in relation to other measures is critical. For a NI checklist, assessing provider technical performance related to visualization of the airway, a critical procedural step, seemed prudent. Not surprisingly, robust correlations existed between checklist scores and measures of airway visualization, CL and POGO. However, there were differences between the airway measures, as dichotomous checklists correlated more strongly with CL than trichotomous checklists, and correlations between dichotomous/ trichotomous ratings and POGO were lower than with CL. This likely relates to improved agreement and reliability with binary ratings in dichotomous checklists, and a limited number of ratings for the ordinal CL scale, compared to the continuous POGO scale [27]. Similar to findings with dichotomous versus trichotomous checklists, having a discrete number of choices when assessing airway visualization confers more precision, and thus, results in stronger correlations.

### Relationship between checklist score and EPA assessment

Previous authors have reported comparisons of GSA scores on simulation-based assessments to EPA ratings [28, 29]. This may provide additional data for EPA assignment, potentially identifying thresholds for entrustment. In our study of two versions of an observational assessment tool containing PSC, GSA, and EPA, the total dichotomous and trichotomous checklist scores increased as the EPA score increased, suggesting that a provider’s checklist score on a simulated NI might be utilized in determining an EPA assessment. For example, based on the current study, in order to be permitted to attempt a clinical NI attempt under direct observation (EPA level 2), a provider would need to score above 10 points on the dichotomous checklist or 22 points on the trichotomous checklist. Similarly, to supervise other providers (EPA level 5), an individual would need to score above 14 points on the dichotomous checklist or 29 points on the trichotomous checklist. Unfortunately, using these results, there is a narrower score range between other EPA categories with overlap of the standard deviations, so it would be challenging to utilize scores on either of these tools to discriminate between providers in the middle of the EPA scale range.

### Selection of Observational Assessment Tool based upon level of training

Consistent with findings from other studies evaluating the impact of experience on performance on observational assessment tools [30, 31], this study demonstrated that the most experienced providers (attending physicians, > 100 clinical intubations) earned lower scores on both checklist versions, but performed well on GSAs, which permit holistic assessment of a procedure. While it may seem counterintuitive, this phenomenon may be explained by an expert’s ability to quickly appraise a situation and skip over certain elements in procedural planning and preparation, perhaps because experience has increased their confidence or, alternatively, due to infrequent procedural opportunities. In contrast, novices tend to rely heavily on granular aspects of procedural technique. While expert providers may fail to see negative consequences of eliminating certain procedural elements, if a procedure is unexpectedly difficult or complicated, they may find themselves suboptimally prepared. Strict adherence to checklists has been advocated for in numerous high-risk fields, including aviation [32], nuclear power [33], and surgery [34]. It seems prudent for providers performing procedures to standardize preparation to optimize patient safety.

### Limitations

This study was conducted in a convenience sample of providers at a single, academic Level IV NICU at a large medical center in New England, United States. These findings may not be generalizable to other settings, including lower-acuity or non-academic centers, or centers in other geographic areas. Since there were fewer individuals in between the novice and very experienced extremes, this may limit the description of NI technical skills across the wider spectrum of providers. The APP group contained individuals with heterogenous experience, making classification of performance for this group more challenging than for those along the physician training spectrum. As with any simulation-based assessment, the fidelity of the mannequin and ability to transfer skills to clinical situations must be considered. Although raters were not provided information about the experience level of participants, it is possible that they may have made inferences about an individual’s experience level based upon physical characteristics. Additionally, it is conceivable that having the raters complete the checklist prior to the GSA and EPA may have influenced their scores on those subsequent measures. Finally, lack of a consistent definition for “procedural competency” continues to hamper assessments and decisions on entrustment.

### Future directions

Assessing on a larger scale how dichotomous and trichotomous checklists perform for formative and summative assessments, both during simulated and clinical intubation attempts, would be valuable. Kuijpers et al.recently published simulation-based study on a trichotomous NI checklist, noting that additional rating options are valuable for formative assessments [35]. Trainee perspectives on added value of trichotomous checklists for formative feedback should be considered.

## Conclusions

In a simulation-based study, there was no difference in ability to discriminate amongst providers at different levels of NI experience using dichotomous or trichotomous checklists when compared to GSA, EPA and airway visualization assessments. The dichotomous checklist conferred benefit in better inter-rater agreement and, therefore, may be preferable for summative assessment purposes. Additional detail provided by the trichotomous checklist may be better suited for formative assessment. Supplementing or substituting the granular detail provided by checklists with general impressions from a GSA or EPA may be considered in time-limited situations (preventing completion of a lengthy checklist) or depending on provider level (ie, rating experts with a GSA may be desired, versus completing an entrustment assessment for a trainee). Therefore, educators should strongly consider the purpose of assessment to inform selection of the optimal assessment tool(s).

## Supplementary Information

**Additional file 1: Appendix A.** INSPIRE Checklist for Neonatal Endotracheal Intubation (Trichotomous).

**Additional file 2: Appendix B.** INSPIRE Checklist for Neonatal Endotracheal Intubation (Dichotomous).

**Additional file 3: Appendix C.** Neonatal Intubation: Trichotomous Checklist Raters’ Guide.

**Additional file 4: Appendix D.** Neonatal Intubation: Dichotomous Checklist Raters’ Guide.

**Additional file 5.** Neonatal Endotracheal Intubation Simulation-Based Observational Assessments: Rater Training and Calibration Materials.

## Data Availability

All data generated or analysed during this study are included in this published article [and its supplementary information files].

## Abbreviations

APP: Advanced pediatric provider
CL: Cormack-Lehane score
EPA: Entrustable professional activities assessment
GSA: Global skills assessment
NICU: Neonatal intensive care unit
POGO: Percent of glottic opening
