# Supplementary material for: Comparison of a dichotomous versus trichotomous checklist for neonatal intubation
Source: BMC Med Educ. 2022 Aug 26;22:645. doi: 10.1186/s12909-022-03700-4 (PMC9419414; doi:10.1186/s12909-022-03700-4)
Supplement: Supplementary file 1 — Additional file 1: Appendix A. INSPIRE Checklist for Neonatal Endotracheal Intubation (Trichotomous). [file 12909_2022_3700_MOESM1_ESM.docx]

**Appendix A.**

**INSPIRE Checklist for Neonatal Endotracheal Intubation (Trichotomous)**

**Learner:**_____________________________ **Learner level:**_________________ **Evaluator**:_______________________

**Context in which procedure performed**:  *Simulation*  *Clinical* **Difficulty level:**  *Normal*  *Difficult*

**Location of procedure:**  NICU  PICU  Pedi ED  OR  Other: ____________

**Describe situation:**______________________________________________________________________________

|  | | | **Done independently.**  **Done correctly.**  **No prompts.** | | **Done with prompt.**  **Done partially.** | **Not done.**  **Done incorrectly.** | |  |
| --- | --- | --- | --- | --- | --- | --- | --- | --- |
|  | | | **2 points** | | **1 point** | **0 points** | | **Not needed/ indicated** |
| **Verbalizes the *indications* for procedure?** | | |  | |  |  | |  |
| **Verbalizes the risks and/ or c*ontraindications* for procedure?** | | |  | |  |  | |  |
| **Verbalizes appropriate *planning* for the procedure (identifies risk factors for difficult intubation, including patient history, anatomic features, and physiologic instability)?** | | |  | |  |  | |  |
| **Verbalizes AND demonstrates appropriate *preparation of equipment* for procedure (including standard intubation equipment, as well as adjunct devices if concern for difficult airway)?** | | |  | |  |  | |  |
| **Requests/ verifies that *appropriate personnel are present* for procedure, including team leader (separate from airway provider) nursing, respiratory therapy, and potentially ENT/ anesthesia if concerns for difficult airway** | | |  | |  |  | |  |
| **Obtains/ verifies consent for elective intubation, identifies patient and performs a time-out?** | | |  | |  |  | |  |
| **Procedural steps completed?** | | | | | | | | |
| 1. **Chooses appropriate size and type of ET tube (if utilized, inserts stylet appropriately)** | | |  | |  |  | |  |
| 1. **Performs equipment check (ensures that laryngoscope, suction, ET tube (with additional tubes/ sizes available), CO2 detector, BVM and monitoring devices), are assembled, available & in working order before commencing procedure** | | |  | |  |  | |  |
| 1. **Demonstrates the appropriate use of sedation/ pre-medication (including possible use of paralytics/ atropine) before commencing procedure** | | |  | |  |  | |  |
| 1. **Performs preoxygenation (method depends on area of practice/ patient population) before commencing procedure** | | |  | |  |  | |  |
| 1. **Demonstrates appropriate positioning of patient (utilizing proper bed height, head position, shoulder roll, and c-spine precautions (if appropriate))** | | |  | |  |  | |  |
| 1. **Employs appropriate technique to open mouth prior to inserting blade** | | |  | |  |  | |  |
| 1. **Demonstrates smooth blade insertion using left hand (must use left hand)** | | |  | |  |  | |  |
| 1. **Demonstrates appropriate technique to lift handle of laryngoscope forward (does not pivot handle, and utilizes smooth movement)** | | |  | |  |  | |  |
| 1. **Demonstrates appropriate technique to visualize vocal cords (understands how to manipulate blade, requests other maneuvers to see cords if necessary) and states when they have achieved view of glottis** | | |  | |  |  | |  |
| 1. **Demonstrates appropriate use of suction (if needed)** | | |  | |  |  | |  |
| 1. **Demonstrates appropriate insertion of ET tube using one smooth motion** | | |  | |  |  | |  |
| 1. **Demonstrates insertion of ET tube to appropriate depth (checks position at level of cords and lip), and verbalizes final position of ETT at lip** | | |  | |  |  | |  |
| 1. **Employs appropriate techniques to confirm correct placement of ET tube by primary (auscultation or confirmation of bilateral chest rise) and secondary (qualitative or quantitative EtCO2 detection) methods** | | |  | |  |  | |  |
| **Procedure successful?**  **Success= *ET tube was placed through the cords (verified using VL OR independent instructor DL OR bilateral chest rise with PPV) on first attempt with <30 seconds of non-ventilated time*** | | |  | |  |  | |  |
| **Demonstrates ability to troubleshoot during procedure when prompted by facilitator (if necessary)?**  **P***lease describe: ­­­­__________________________________* | | |  | |  |  | |  |
| **If complications encountered, please describe:** | | |  | | | | | |
| **Performs appropriate aftercare (Secures ET tube, confirms placement with CXR, selects ventilator settings)** | | |  | |  |  | |  |
| **Global assessment of procedural skill** (circle one) | | | | | | | | |
| **1** | **2** | **3** | | **4** | | | **5** | |
| **Novice**  *Early stage of learning where skills are learned through imitation and/or trial and error. More than 2 prompts* | **Advanced Beginner**  *Skills have become habitual and the movements can be performed with some confidence and proficiency. 1 to 2 prompts* | **Competent**  *Proficiency is indicated by a quick, accurate, and highly coordinated performance. No prompts, no errors, under normal circumstances.* | | **Proficient**  *Skills are well developed and the individual can modify movement patterns to address difficult situations. No prompts, no errors, under difficult circumstances.* | | | **Expert**  *Individual able to create new movement patterns to address a unique situation, or specific problem*. | |

**Entrustable Professional Activity (EPA) assessment:**

Ready to observe the activity only, not ready to perform procedure on patient even with direct supervision*

Ready to perform procedure with direct supervision present in the room

Ready to perform the procedure with supervision available within minutes

Ready to perform procedure without direct supervision (i.e., under clinical oversight)

Ready to provide supervision to juniors learning the procedure

* If learner/ trainee not competent to perform procedure please refer for remedial simulation training
