# Supplementary material for: Comparison of a dichotomous versus trichotomous checklist for neonatal intubation
Source: BMC Med Educ. 2022 Aug 26;22:645. doi: 10.1186/s12909-022-03700-4 (PMC9419414; doi:10.1186/s12909-022-03700-4)
Supplement: Supplementary file 3 — Additional file 3: Appendix C. Neonatal Intubation: Trichotomous Checklist Raters’ Guide. [file 12909_2022_3700_MOESM3_ESM.docx]

**Appendix C:**

**Neonatal Intubation: Trichotomous Checklist Raters’ Guide**

|  | **Done independently.**  **Done correctly.**  **No prompts.** | **Done with prompt.**  **Done partially.** | **Not done.**  **Done incorrectly.** |
| --- | --- | --- | --- |
|  | **2 points** | **1 point** | **0 points** |
| **Verbalizes the *indications* for procedure?** | Clearly states reason for intubation aloud (ie, “prolonged apnea” or “HR below 100 bpm with effective PPV”) | More disorganized, vague statement about why intubation planned | Indication not stated aloud |
| **Verbalizes the risks and/ or c*ontraindications* for procedure?** | Clearly discusses potential risks RE: worsening physiologic instability, other options for airway management (if applicable) | More disorganized, not clearly addressed | Risks not stated aloud |
| **Verbalizes appropriate *planning* for the procedure (identifies risk factors for difficult intubation, including patient history, anatomic features, and physiologic instability)?** | Discusses all 3 items listed here:  1. history of prior intubations  2. anatomic features  3. physiologic instability | Discusses 1-2 items listed for “full credit” | Does not discuss any of the planning items listed |
| **Verbalizes AND demonstrates appropriate *preparation of equipment* for procedure (including standard intubation equipment, as well as adjunct devices if concern for difficult airway)?** | Establishes that all equipment necessary for intubation is present and in working order, (and discusses additional equipment to consider for difficult airway) prior to commencing procedure | Finds and checks some, but not all, equipment prior to commencing procedure | Does not find & check any equipment prior to commencing procedure |
| **Requests/ verifies that *appropriate personnel are present* for procedure, including team leader (separate from airway provider) nursing, respiratory therapy, and potentially ENT/ anesthesia if concerns for difficult airway** | Requests/ verifies that additional providers (RN, RT at minimum) are present, and assigns them positions | Requests/ verifies that either RN/ RT if present; may not specifically assign tasks/ positions | Does not request/ verify the presence of other providers prior to commencing procedure |
| **Obtains/ verifies consent for elective intubation, identifies patient and performs a time-out?** | Must confirm that consent has been obtained, and conduct a “time out” prior to commencing procedure | Completes *either* confirmation of consent, or time out, *but not both* | Does not complete either step (confirmation of consent + time out) |
|  | | | |
| 1. **Chooses appropriate size and type of ET tube (if utilized, inserts stylet appropriately)** | Choose 3-5, un-cuffed ETT for 3kg infant in this study; If used, stylet should terminate ABOVE the side hole & end hole of ETT | Choose 3-0 ETT; unsure of size needed & asks confederate RN | Chooses 2-5 ETT;  Stylet protrudes from side-hole or end-hole of ETT |
| 1. **Performs equipment check (ensures that laryngoscope, suction, ET tube (with additional tubes/ sizes available), CO2 detector, BVM and monitoring devices), are assembled, available & in working order before commencing procedure** | Identifies and checks all pieces of equipment listed here:  Miller 1 Laryngoscope  -*Assembles*  *-Checks light source*  Suction  ETT (additional sizes)  Stylet  CO2 detector  BVM + face mask  Monitoring device  ***** Needs to check prior to starting procedure*** | Identifies and checks some, but not all, pieces of equipment listed prior to starting procedure | Does not identify and check any equipment prior to staring procedure |
| 1. **Demonstrates the appropriate use of sedation/ pre-medication (including possible use of paralytics/ atropine) before commencing procedure** | 1. Confirms IV access  2. Discusses pre-medication as per unit guidelines… DOES NOT SPECIFICALLY NEED TO STATE MEDICATION DOSES**  3. Gives premedication prior to beginning procedure | 1. Unsure of dosing/ types of medication, but asks confederate RN for assistance (and is directed to unit guidelines/ protocol)  2. Gives premedication prior to beginning procedure | Does not utilize premedication prior to elective intubation |
| 1. **Performs preoxygenation (method depends on area of practice/ patient population) before commencing procedure** | Providers should provide PPV to manikin & comment upon saturations/ stability prior to beginning procedure. *(Since the focus of this checklist is NOT PPV, poor technique will not count against the score for this item.)* | PPV given, but does not comment upon saturations/ stability prior to beginning procedure | Does not provide PPV prior to starting procedure |
| 1. **Demonstrates appropriate positioning of patient (utilizing proper bed height, head position, shoulder roll, and c-spine precautions (if appropriate))** | Provider must physically demonstrate positioning into the “sniffing” position—must move head/ body; bed height must be appropriate (at provider’s waist level), and railing at top of bed should be down; shoulder roll is acceptable but not mandatory | Provider physically demonstrates positioning into sniffing position, but does not adjust bed height or railing (if necessary) | Does not position of patient prior to starting procedure |
| 1. **Employs appropriate technique to open mouth prior to inserting blade** | Provider must use their fingers to “open” manikin’s mouth (rather than just insert blade) | Clumsy or disorganized technique for opening mouth | Does not use fingers to “open” manikin’s mouth |
| 1. **Demonstrates smooth blade insertion using left hand (must use left hand)** | Uses left hand, and demonstrates careful gentle insertion | Uses left hand, more clumsy & disorganized technique | Uses right hand, extremely rough |
| 1. **Demonstrates appropriate technique to lift handle of laryngoscope forward (does not pivot handle, and utilizes smooth movement)** | Once cords visualized, “lifts” handle at a 45 degree angle; avoids “rocking” back on gums/ lips | Some minimal amount of “rocking” | Significant “rocking” back on gums/ lips |
| 1. **Demonstrates appropriate technique to visualize vocal cords (understands how to manipulate blade, requests other maneuvers to see cords if necessary) and states when they have achieved view of glottis** | Carefully adjusts tip of blade to visualize glottic opening; may request cricoid pressure;  **Must verbalize when view of glottis/ cords achieved** | Clumsy or disorganized movements to identify view of glottic opening;  Does not verbalize when view of glottis/ cords achieved | Very rough movements utilized to visualize glottic opening;  Seems unfamiliar with techniques to adjust view;  Does not verbalize when view of glottis/ cords achieved |
| 1. **Demonstrates appropriate use of suction (if needed)** | Unless secretions are specifically mentioned by the instructor, these should not be present in the airway. **Reference rating in this case should be “non-applicable”** | - | Use of suction during the procedure (unless secretions specifically noted by instructor) would be inappropriate, as no secretions are present in manikin’s airway |
| 1. **Demonstrates appropriate insertion of ET tube using one smooth motion** | Smooth controlled technique utilized; ETT should be inserted in side of mouth (not down bevel of laryngoscope) | Less controlled insertion of ETT; inserted down bevel of laryngoscope | Very rough, clumsy insertion of ETT |
| 1. **Demonstrates insertion of ET tube to appropriate depth (checks position at level of cords and lip), and verbalizes final position of ETT at lip** | ETT inserted to appropriate depth (either 9-10cm at lip in this 3kg patient) initially, and position confirmed aloud verbally | ETT initially inserted to inappropriate depth, but repositioned appropriately; final position confirmed aloud verbally | ETT inserted to inappropriate depth and NOT repositioned; final position not confirmed aloud verbally |
| 1. **Employs appropriate techniques to confirm correct placement of ET tube by primary (auscultation or confirmation of bilateral chest rise) and secondary (qualitative or quantitative EtCO2 detection) methods** | Utilizes both auscultation/ assessment of chest rise & CO2 detection to confirm correct placement of ETT | Either uses auscultation/ assessment of chest rise **OR** CO2 detection to confirm correct placement *(but not both)* | Does not use any techniques to confirm correct placement of ETT |
| **Procedure successful?**  **Success= *ET tube was placed through the cords (verified using VL OR independent instructor DL OR bilateral chest rise with PPV) on first attempt with <30 seconds of non-ventilated time*** | Must satisfy all criteria listed here for “successful” procedure:  1.Placed through cords (confirmed by chest rise, which would be noted by confederate RN)  2. NEEDS TO BE SUCCESSFUL ON FIRST ATTEMPT**  **Attempt= any placement of laryngoscope blade in mouth**  3. Non-ventilated time (starting from insertion of blade until PPV begins through ETT) should be <30 seconds | ETT successfully placed though cords on FIRST ATTEMPT but non-ventilated time > 30 seconds | Any of the following would make the attempt “unsuccessful”:  1. Unsuccessful attempt to place ETT through cords  2. Requires more than one “attempt” (attempt= any placement of laryngoscope blade in mouth) |
| **Demonstrates ability to troubleshoot during procedure when prompted by facilitator (if necessary)?**  **P***lease describe: ­­­­__________________________________* | For this study, this should be interpreted as troubleshooting for issues that arise for patient instability or equipment malfunction, NOT to compensate for suboptimal procedural technique… therefore, for all of the items on the videos you will be rating, this should be “non-applicable” | For this study, this should be interpreted as troubleshooting for issues that arise for patient instability or equipment malfunction, NOT to compensate for suboptimal procedural technique… therefore, for all of the items on the videos you will be rating, this should be “non-applicable” | For this study, this should be interpreted as troubleshooting for issues that arise for patient instability or equipment malfunction, NOT to compensate for suboptimal procedural technique… therefore, for all of the items on the videos you will be rating, this should be “non-applicable” |
| **If complications encountered, please describe:** | N/A | | |
| **Performs appropriate aftercare (Secures ET tube, confirms placement with CXR, selects ventilator settings)** | Performs ALL of the following:  1. Asks RN to secure ETT  2. Requests CXR  3. Alludes to selection of ventilator settings with RT (does not need to specifiy any particular settings) | Performs 1-2 “aftercare” items, but not all three | Does not perform any “aftercare” items |
