# Supplementary material for: Comparison of a dichotomous versus trichotomous checklist for neonatal intubation
Source: BMC Med Educ. 2022 Aug 26;22:645. doi: 10.1186/s12909-022-03700-4 (PMC9419414; doi:10.1186/s12909-022-03700-4)
Supplement: Supplementary file 4 — Additional file 4: Appendix D. Neonatal Intubation: Dichotomous Checklist Raters’ Guide. [file 12909_2022_3700_MOESM4_ESM.docx]

**Appendix D:**

**Neonatal Intubation: Dichotomous Checklist Raters’ Guide**

|  | **Done correctly** | **Done incorrectly or Not done** | **Not needed or applicable** |
| --- | --- | --- | --- |
|  | **1 points** | **0 points** | **N/A** |
| **Verbalizes the *indications* for procedure?** | Clearly states reason for intubation aloud (ie, “prolonged apnea” or “HR below 100 bpm with effective PPV”) | Indication not stated aloud in a clear and organized manner |  |
| **Verbalizes the risks and/ or c*ontraindications* for procedure?** | Clearly discusses potential risks RE: worsening physiologic instability, other options for airway management (if applicable) | Risks not stated aloud in a clear and organized manner |  |
| **Verbalizes appropriate *planning* for the procedure (identifies risk factors for difficult intubation, including patient history, anatomic features, and physiologic instability)?** | Discusses all 3 items listed here:  1. history of prior difficult intubations (if applicable)  2. anatomic features  3. physiologic instability | Does not discuss any of the planning items listed |  |
| **Verbalizes AND demonstrates appropriate *preparation of equipment* for procedure (including standard intubation equipment, as well as adjunct devices if concern for difficult airway)?** | Establishes that all equipment necessary for intubation is present and in working order, (and discusses additional equipment to consider for difficult airway) prior to commencing procedure | Does not find & check all necessary equipment prior to commencing procedure |  |
| **Requests/ verifies that *appropriate personnel are present* for procedure, including team leader (separate from airway provider) nursing, respiratory therapy, and potentially ENT/ anesthesia if concerns for difficult airway** | Requests/ verifies that additional providers (RN, RT at minimum) are present, and assigns them roles | Does not request/ verify the presence of other providers and assign specific roles prior to commencing procedure |  |
| **Obtains/ verifies consent for elective intubation, identifies patient and performs a time-out?** | Must confirm that consent has been obtained, and conduct a “time out” prior to commencing procedure | Does not complete both steps (confirmation of consent + time out) prior to commencing procedure |  |
| 1. **Chooses appropriate size and type of ET tube (if utilized, inserts stylet appropriately)** | Choose 3-5 or 4-0, un-cuffed ETT for 3kg infant in this study; If used, stylet should terminate ABOVE the side hole & end hole of ETT | Chooses 3-0 or 2-5 ETT;  Stylet protrudes from side-hole or end-hole of ETT |  |
| 1. **Performs equipment check (ensures that laryngoscope, suction, ET tube (with additional tubes/ sizes available), CO2 detector, BVM and monitoring devices), are assembled, available & in working order before commencing procedure** | Identifies and checks all pieces of equipment listed here:  1. Miller 1 Laryngoscope  -*Assembles*  *-Checks light source*  2. Suction  3. ETT ( plus additional sizes)  4. Stylet  5. CO2 detector  6. BVM + face mask  7. Monitoring devices  ***** Needs to check prior to starting procedure*** | Does not identify and check all listed equipment prior to staring procedure |  |
| 1. **Demonstrates the appropriate use of sedation/ pre-medication (including possible use of paralytics/ atropine)** | 1. Confirms IV access  2. Discusses pre-medication as per unit guidelines… DOES NOT SPECIFICALLY NEED TO STATE MEDICATION DOSES**  **** For premedication, providers do not need to know specific doses or types of medications, as long as they verbalize that they will reference their unit’s policy/ guidelines for intubation premedication & order accordingly****  3. Gives premedication prior to beginning procedure | Does not utilize appropriate premedication prior to elective intubation;  **** If providers are unsure about the dosing/ meds & are given a prompt regarding the unit’s policy/ guidelines, they should not get credit for this item.** |  |
| 1. **Performs preoxygenation (method depends on area of practice/ patient population)** | Providers should provide PPV to manikin & comment upon saturations/ stability prior to beginning procedure. *(Since the focus of this checklist is NOT PPV, poor technique will not count against the score for this item.)* | Does not provide PPV prior to starting procedure, or does not comment upon saturations/ stability prior to beginning procedure |  |
| 1. **Demonstrates appropriate positioning of patient (utilizing proper bed height, head position, shoulder roll, and c-spine precautions (if appropriate))** | Provider must physically demonstrate positioning into the “sniffing” position—must move head/ body; bed height must be appropriate (at provider’s waist level), and railing at top of bed should be down; shoulder roll is acceptable but not mandatory | Does not physically adjust position of patient prior to starting procedure (including bed height, railings) |  |
| 1. **Employs appropriate technique to open mouth prior to inserting blade** | Provider must use their fingers to “open” manikin’s mouth (rather than just insert blade) | Does not physically use fingers to “open” manikin’s mouth |  |
| 1. **Demonstrates smooth blade insertion using left hand (must use left hand)** | Uses left hand, and demonstrates careful gentle insertion | Uses right hand, or has extremely rough movements |  |
| 1. **Demonstrates appropriate technique to lift handle of laryngoscope forward (does not pivot handle, and utilizes smooth movement)** | Once cords visualized, “lifts” handle at a 45 degree angle; avoids “rocking” back on gums/ lips | Significant “rocking” back on gums/ lips |  |
| 1. **Demonstrates appropriate technique to visualize vocal cords (understands how to manipulate blade, requests other maneuvers to see cords if necessary) and states when they have achieved view of glottis** | Carefully adjusts tip of blade to visualize glottic opening; may request cricoid pressure;  **Must verbalize when view of glottis/ cords achieved** | Very rough movements utilized to visualize glottic opening;  Seems awkward or unfamiliar with techniques to adjust view;  Does not verbalize when view of glottis/ cords achieved |  |
| 1. **Demonstrates appropriate use of suction (if needed)** | Unless secretions are specifically mentioned by the instructor, these should not be present in the airway. **Reference rating in this case should be “non-applicable”** | Use of suction during the procedure (unless secretions specifically noted by instructor) would be inappropriate, as no secretions are present in manikin’s airway | Unless secretions are specifically mentioned by the instructor, these should not be present in the airway. **Reference rating in this case should be “non-applicable”** |
| 1. **Demonstrates appropriate insertion of ET tube using one smooth motion** | Smooth controlled technique utilized; ETT should be inserted in side of mouth (not down bevel of laryngoscope) | Very rough, clumsy insertion of ETT; requires more than one attempt to pass ETT |  |
| 1. **Demonstrates insertion of ET tube to appropriate depth (checks position at level of cords and lip), and verbalizes final position of ETT at lip** | ETT inserted to appropriate depth (either 9-10cm at lip in this 3kg patient) initially (or readjusted immediately after evaluating placement at lip), and position confirmed aloud verbally | ETT inserted to inappropriate depth and NOT repositioned; final position not confirmed aloud verbally |  |
| 1. **Employs appropriate techniques to confirm correct placement of ET tube by primary (auscultation or confirmation of bilateral chest rise) and secondary (qualitative or quantitative EtCO2 detection) methods** | Utilizes both auscultation/ assessment of chest rise & CO2 detection to confirm correct placement of ETT | Does not use both of the listed techniques to confirm correct placement of ETT |  |
| **Procedure successful?**  **Success= *ET tube was placed through the cords (verified using VL OR independent instructor DL OR bilateral chest rise with PPV) on first attempt with <30 seconds of non-ventilated time*** | Must satisfy all criteria listed here for “successful” procedure:  1.Placed through cords (confirmed by chest rise, which would be noted by confederate RN)  2. NEEDS TO BE SUCCESSFUL ON FIRST ATTEMPT**  **Attempt= any placement of laryngoscope blade in mouth**  3. Non-ventilated time (starting from insertion of blade until PPV begins through ETT) should be <30 seconds | Does not satisfy all 3 criteria for “successful” procedure |  |
| **Demonstrates ability to troubleshoot during procedure when prompted by facilitator (if necessary)?**  Please describe*: __________________________________* | For this study, this should be interpreted as troubleshooting for issues that arise for patient instability or equipment malfunction, NOT to compensate for suboptimal procedural technique… therefore, for all of the items on the videos you will be rating, this should be “non-applicable” | For this study, this should be interpreted as troubleshooting for issues that arise for patient instability or equipment malfunction, NOT to compensate for suboptimal procedural technique… therefore, for all of the items on the videos you will be rating, this should be “non-applicable” | For this study, this should be interpreted as troubleshooting for issues that arise for patient instability or equipment malfunction, NOT to compensate for suboptimal procedural technique… therefore, for all of the items on the videos you will be rating, this should be “non-applicable” |
| **If complications encountered, please describe:**  *__________________________________* |  |  |  |
| **Performs appropriate aftercare (Secures ET tube, confirms placement with CXR, selects ventilator settings)** | Performs ALL of the following:  1. Asks RN to secure ETT  2. Requests CXR  3. Alludes to selection of ventilator settings with RT (does not need to specifiy any particular settings) | Does not perform all 3 of the listed steps for aftercare |  |
