# Supplementary material for: Comparison of a dichotomous versus trichotomous checklist for neonatal intubation
Source: BMC Med Educ. 2022 Aug 26;22:645. doi: 10.1186/s12909-022-03700-4 (PMC9419414; doi:10.1186/s12909-022-03700-4)
Supplement: Supplementary file 5 — Additional file 5. Neonatal Endotracheal Intubation Simulation-Based Observational Assessments: Rater Training and Calibration Materials. [file 12909_2022_3700_MOESM5_ESM.docx]

**Neonatal Endotracheal Intubation Simulation-Based Observational Assessments:**

**Rater Training and Calibration Materials**

The purpose of this resource is to introduce experts in neonatal endotracheal intubation (NETI) to the development and application of a checklist and global rating scale (GRS) for simulation-based observational assessments of NETI that was developed through the International Network for Simulation-Based Pediatric Innovation, Research, and Education (INSPIRE) network. Expert raters using this resource will better understand the process of evaluating simulated NETI performance using these tools.

**Outline**

1. Objectives
2. Process for Rater Training and Calibration
3. Overview and Background
4. Preliminary Work in Checklist Development
5. Materials
   1. NETI Procedural Skills Checklists (Dichotomous/ Trichotomous)
   2. Global Rating Scale
6. References

**Objectives for expert raters:**

1. Articulate the process by which the INSPIRE NETI procedural skills checklist and GRS were developed.
2. Describe criteria for rating a simulation-based NETI attempt using the INSPIRE NETI checklist and GRS.
3. Demonstrate appropriate application of the INSPIRE NETI checklist and GRS (as compared to the “reference standard” scores), and refine scoring accuracy through process of feedback and calibration.

**Process for Rater Training & Calibration:**

1. Experts should review materials for observational assessment of NETI (INSPIRE procedural skills checklist & GRS).
2. As part of the rater training course, experts should view the calibration videos in sequence and rate them using the INSPIRE tools provided.
3. Following the expert rating of each video, group discussion will occur regarding why providers were scored the way they were with comparison to the scores given by the checklist development team (“reference standard” scores).
4. This process will be repeated for subsequent videos until inter-rater reliability (IRR) level acceptable.

**Overview of INSPIRE Neonatal Endotracheal Intubation Simulation-Based Observational Assessment Tools**

This project is the product of ~1.5 years of work by our group to develop a checklist and global rating scale (GRS) to be utilized in simulation-based NETI procedures as part of the procedural training initiative of the INSPIRE research network. These materials will be utilized as part of the six-step procedural training “package,” which includes preparation (LEARN), demonstrations (SEE), Simulation-based training (PRACTICE), summative assessment on a simulator (PROVE), clinical performance (DO), and continued training and performance (MAINTAIN).^1^ The overarching goal of the INSPIRE Package is to improve clinical success rates and outcomes of pediatric procedures.

The purpose of the materials in this rater training sessions is to instruct experts in NETI to utilize the developed observational assessment tools. This instruction is being performed as part of a study to assess for various sources of validity evidence for these assessment tools. Ultimately, these tools will be utilized in the PRACTICE and PROVE steps listed in the paradigm above. The simulated environment provides a means for trainees to achieve a specific competency level via practice without affecting live patients.^2^

This intervention is competency-based and parallels the American Council for Graduate Medical Education (ACGME) milestones and entrustable professional activities (EPAs) by proving mastery prior to performing NETI clinically.

**Background**

Neonatal endotracheal intubation (NETI) is a critically important procedure for providers working in the delivery room and neonatal intensive care unit (NICU). Approximately 10% of infants require resuscitation at birth, including possible NETI.^3^ Sub-optimal NETI technique has been associated with multiple or prolonged procedural attempts, direct airway injury, patient deterioration, and even death.^4-6^

NETI performance amongst pediatric trainees has been poor.^7,8^ Reported success rates for pediatric residents are low, ranging from 20-60%.^7-12^ A recent study by Haubner et al. in five academic Neonatal Intensive Care Units (NICUs) reported an overall per-attempt success rate of only 44% for all providers.^13^ The highest success rates were in attending physicians (72%) and senior neonatal fellows (70%).^13^ Success rates for interns were only 20%, and no significant improvement was noted with advancing years of residency training.^13^ Thus, there is an inherent risk to patients who are intubated by junior trainees, as the likelihood of success is significantly lower than that expected with an experienced provider. As the number of attempts to successfully secure the airway increases, so does the likelihood of adverse patient outcomes.

These low success rates are likely multifactorial, and include recent restrictions on trainee work hours and number of ICU rotations,^14^ increased presence of mid-level providers (nurse practitioners and physician assistants,^15^ changes in management guidelines for infants with meconium-stained amniotic fluid,^16^ and increased usage of non-invasive ventilation strategies.^17^ Given these challenges, it is unlikely that the majority of residents will be competent to safely perform NETI after graduation despite the fact that this is listed as a requirement for completion of pediatric residency training by the ACGME. However, this skill remains important for pediatricians,^18, 19^ particularly those practicing in rural settings^20,21^ or entering fellowship training in a critical care subspecialty.^22^

Educational strategies to improve NETI performance are necessary. Simulation has been utilized frequently in procedural skills training in pediatrics and neonatology.^23^ Although improved performance in a simulated setting does not always increase clinical success rates,^24^ previous authors have demonstrated improved clinical performance after rigorous simulation-based training utilizing deliberate practice (DP) and mastery learning (ML).^25, 26^ Immediately following a simulated procedural attempt, providers receive formative feedback directed at improving performance to a pre-defined standard. Learners continue training until the uniform result of skill mastery has been achieved (i.e., when the minimum passing score (MPS) on a valid assessment tool is attained), but the time required to reach this standard is variable. This methodology has improved performance in clinical procedures such as central line placement, dialysis catheter insertion, thoracentesis, Adult Cardiac Life Support (ACLS) performance, and infant lumbar puncture (ILP).^27-36^ Furthermore, in a critical review of the simulation-based ML literature, McGaghie, Issenberg, Barsuk, & Wayne concluded that ML in medical education results in improvement in such distal outcomes as performance in educational “laboratories” (T1), patient care practices (T2), patient outcomes (T3), and “collateral effects”, including cost savings and impact on other trainees in the health care setting (T4).^37^

In this new era of competency-based medical education,^38,39^ having strategies to objectively rate performance is crucial. Sawyer et al recently developed an evidence-based pedagogical framework for procedural skills training, the Learn-See-Practice-Prove-Do-Maintain (LSPPDM) Model.^1^ During the *Prove* step, it is recommended that learners achieve the minimum passing score (MPS) on an assessment tool with sources of validity evidence during a procedural attempt in a simulation-based mastery learning (ML) session before transitioning to clinical procedural attempts under direct supervision.^1^ Therefore, it would follow that instruments with sources of validity evidence should be developed for each required procedure.

Therefore, due to their critical role in both training and assessment, development of validated and reliable tools is of paramount importance for medical educators training novice providers to perform NETI.

**Preliminary Work/ Development of NETI Procedural Skills Checklist and GRS**

The aim of the current study is to provide multiple sources of validity evidence for a procedural skills checklist and global ratings scale (GRS) for NETI. The checklist utilized for this study was developed through the INSPIRE Research Network (International Network for Simulation-Based Pediatric Innovation, Research, and Education, <http://inspiresim.com>), in an effort led by the primary investigator of the present study. A group of board-certified pediatric subspecialists (Neonatology, Pediatric Critical Care Medicine, PEM, and Pediatric Anesthesia) reviewed the medical literature, published procedural skills checklists, MedEdPortal, and procedural skills training videos to identify resources for endotracheal intubation. Only one checklist with validity evidence was identified for NETI, which had been developed for nasotracheal intubation by Bismilla, et al.^7^ This checklist had also been previously modified through Delphi technique and used in one clinical study in NETI,^13^ but no further validity testing had been performed. The INSPIRE group re-evaluated the content of the checklist, and reformatted it utilizing a standardized template developed by the network.

Next, a modified Delphi process was utilized to optimize the content of the checklist. Twelve pediatric sub-specialist experts who had not been involved in the design of the initial checklist participated in the Delphi rounds. These individuals represented a variety of specialties (Neonatology, Pediatric Critical Care Medicine, Pediatric Emergency Medicine, Pediatric Otolaryngology, and Pediatric Anesthesiology), as well as a number of geographic areas (United States, Canada, and Japan). All specialists were board-certified or fully licensed, and had been in independent practice for a minimum of five years.

Delphi rounds were conducted via email. Each specialist was instructed to review each step of the trichotomous procedural skills checklist, and rate it on a Likert scale (1- not important to 7- critical). Raters were asked to provide comments on the existing steps of the checklist, and identify additional steps that had not been included. Completed checklists were de-identified and sent to a blinded reviewer for statistical analysis. It had been previously determined that items with a mean score of <4 would be eliminated, and newly identified steps would be added. The resulting checklist was then re-distributed for further review and comments. The process continued for a total of two rounds, after which time no additional comments were provided. The initial checklist contained 13 items, and no item scored <4 on average during either round. No additional items were added. However, some of the existing items were modified. The goal Cronbach’s alpha was 0.7-0.9. For the first draft, Cronbach’s alpha was 0.727 with ICC of 0.78. In the second draft, Cronbach’s alpha was 0.862. The checklist and GRS are provided in Appendices A (dichotomous version) and B (trichotomous version).

**Procedure for Rater Training and Calibration**

Four board-certified neonatologists employed at academic teaching centers other than Yale University and in practice for a minimum of five years will serve as raters. During a dedicated webinar, raters will be introduced to the purpose of the study. They will be given information about the development of the NETI procedural skills checklist and GRS and instructed on how these assessment tools should be used to rate performance. Raters will be calibrated during a process of scoring three videos of providers at varying levels of skill performing NETI on a simulator. The training videos were developed by a neonatologist at the University of Washington. They include an external view of the subject’s NETI performance along with a simultaneous recording from a Storz CMAC Videolaryngoscope (VL) (Karl Storz Endoskope, Tuttingen, Germany), which displays a real-time view of the airway during the NETI attempt. Raters will score these videos using the checklist and GRS, and then participate in a discussion comparing their ratings to the “reference standard” ratings that have been pre-determined through discussion of the study investigators. Through this process of rating and feedback, the trained rater’s overall checklist scores should begin to closely approximate the “gold standard” scores. The calibration process will continue until the inter-rater reliability is >0.8, appropriate for this moderate stakes assessment.^40^

Two additional board-certified neonatologists in practice for >5 years will serve as blinded raters for the airway visualization obtained from the VL recordings. They will also undergo a rater training and calibration session prior to the start of the study based upon video recorded examples that approximate various degrees of airway visualization corresponding to the CL scale.

Refer to Appendices A, B, C, and D for the dichotomous and trichotomous checklists, as well as description of performance for each item.

**Notes for raters:**

1. The intubations you will be rating during this calibration session are all for an urgent intubation of a patient who was previously admitted to the NICU (ie, not in the delivery room).
2. The checklist is intended to rate the team responsible for the intubation (not only the individual managing the airway).
3. You will be rating each of 3 scenarios using both a dichotomous and trichotomous skills checklist, a Global Ratings Scale (GRS), and an Entrustable Professional Activities (EPA) measure.

**Notes for specific checklist items:**

1. For “appropriate planning of procedure,” should discuss pertinent aspects of patient history, anatomy, and vital signs/ physiologic stability, as well as how these might impact (or be affected by) the intubation procedure.
2. For item #10, unless secretions specifically mentioned by instructor, these should not be present in the manikin’s airway. The reference rating would therefore be N/A, and simulated use of suction in these cases would be incorrect.
3. For item #12, provider would earn 2 points if they do not initially insert the tube into the right mainstem (and confirm appropriate position); 1 point if initially deeply inserted but adjusted subsequently; and 0 points if deeply inserted and not adjusted.
4. In determining if the procedure was successful, please note that the tube needs to be successfully placed on the first attempt.
5. The item on “troubleshooting” should be interpreted as troubleshooting for issues that arise with patient instability or equipment malfunction, NOT to compensate for suboptimal technique.

**References**

1. Sawyer T, White M, Zaveri P, Chang T, Ades A, French H et al. Learn, See, Practice, Prove, Do, Maintain. Academic Medicine. 2015;90(8):1025-1033.
2. Kneebone R. Practice, Rehearsal, and Performance. JAMA. 2009;302(12):1336.
3. Weiner G, Zaichkin J. Textbook of Neonatal Resuscitation. 8th ed. Itasca: American Academy of Pediatrics; 2021.
4. Chen J, Susetio L, Chao C. Oral complications associated with endotracheal general anesthesia. Ma Zui Xue Za Zhi. 1990;28(2):163-169.
5. Easley R, Segeleon J, Haun S, Tobias J. Prospective study of airway management of children requiring endotracheal intubation before admission to a pediatric intensive care unit. Critical Care Medicine. 2000;28(6):2058-2063.
6. Gausche M, Lewis R, Stratton S, Haynes B, Gunter C, Goodrich S et al. Effect of Out-of-Hospital Pediatric Endotracheal Intubation on Survival and Neurological Outcome. JAMA. 2000;283(6):783-790.
7. Bismilla Z, Finan E, McNamara P, LeBlanc V, Jefferies A, Whyte H. Failure of pediatric and neonatal trainees to meet Canadian Neonatal Resuscitation Program standards for neonatal intubation. Journal of Perinatology. 2009;30(3):182-187.
8. Downes K, Narendran V, Meinzen-Derr J, McClanahan S, Akinbi H. The lost art of intubation: assessing opportunities for residents to perform neonatal intubation. Journal of Perinatology. 2012;32(12):927-932.
9. Gozzo Y, Cummings C, Chapman R, Bizzarro M, Mercurio M. Who is performing medical procedures in the neonatal intensive care unit? Journal of Perinatology. 2010;31(3):206-211.
10. Leone T, Rich W, Finer N. Neonatal intubation: Success of pediatric trainees. The Journal of Pediatrics. 2005;146(5):638-641.
11. O'Donnell C, Kamlin C, Davis P, Morley C. Endotracheal Intubation Attempts During Neonatal Resuscitation: Success Rates, Duration, and Adverse Effects. Pediatrics. 2006;117(1):e16-e21.
12. Sagarin M, Chiang V, Sakles J, Barton E, Wolfe R, Vissers R et al. Rapid sequence intubation for pediatric emergency airway management. Pediatric Emergency Care. 2002;18(6):417-423.
13. Haubner L, Barry J, Johnston L, Soghier L, Tatum P, Kessler D et al. Neonatal intubation performance: Room for improvement in tertiary neonatal intensive care units. Resuscitation. 2013;84(10):1359-1364.
14. Accreditation Council for Graduate Medical Education, Common Program Requirements for Graduate Medical Education in Pediatrics. <https://www.acgme.org/globalassets/PFAssets/ProgramRequirements/320_Pediatrics_2020.pdf?ver=2020-06-29-162726-647&ver=2020-06-29-162726-647>. Accessed April 8, 2022.
15. Lee H, Rhee C, Sectish T, Hintz S. Changes in Attendance at Deliveries by Pediatric Residents 2000 to 2005. American Journal of Perinatology. 2008;26(02):129-134.
16. Whitfield J, Charsha D, Chiruvolu A. Prevention of Meconium Aspiration Syndrome: An Update and the Baylor Experience. Baylor University Medical Center Proceedings. 2009;22(2):128-131.
17. DeMauro S, Millar D, Kirpalani H. Noninvasive respiratory support for neonates. Current Opinion in Pediatrics. 2014;26(2):157-162.
18. Gaies M, Landrigan C, Hafler J, Sandora T. Assessing Procedural Skills Training in Pediatric Residency Programs. Pediatrics. 2007;120(4):715-722.
19. Gaies M, Landrigan C, Hafler J, Sandora T. Assessing Procedural Skills Training in Pediatric Residency Programs: In Reply. Pediatrics. 2008;121(3):650-651.
20. Feigin R, Drutz J, Smith E, Collins C. Practice Variations by Population: Training Significance. Pediatrics. 1996;98(2):186-190.
21. Wood A, Jones M, Wood J, Pan Z, Parker T. Neonatal Resuscitation Skills Among Pediatricians and Family Physicians: Is Residency Training Preparing for Postresidency Practice?. Journal of Graduate Medical Education. 2011;3(4):475-480.
22. Jones M, McGuinness G. The Future of Pediatric Residency Education: Prescription for More Flexibility. The Journal of Pediatrics. 2009;154(2):157-158.e3.
23. Anderson J, Warren J. Using Simulation to Enhance the Acquisition and Retention of Clinical Skills in Neonatology. Seminars in Perinatology. 2011;35(2):59-67.
24. Finan E, Bismilla Z, Campbell C, LeBlanc V, Jefferies A, Whyte H. Improved procedural performance following a simulation training session may not be transferable to the clinical environment. Journal of Perinatology. 2011;32(7):539-544.
25. Ericsson K. Deliberate Practice and the Acquisition and Maintenance of Expert Performance in Medicine and Related Domains. Academic Medicine. 2004;79(Supplement):S70-S81.
26. McGaghie W, Issenberg S, Cohen E, Barsuk J, Wayne D. Does Simulation-Based Medical Education With Deliberate Practice Yield Better Results Than Traditional Clinical Education? A Meta-Analytic Comparative Review of the Evidence. Academic Medicine. 2011;86(6):706-711.
27. Barsuk J, McGaghie W, Cohen E, Balachandran J, Wayne D. Use of simulation-based mastery learning to improve the quality of central venous catheter placement in a medical intensive care unit. Journal of Hospital Medicine. 2009;4(7):397-403.
28. Barsuk J, Cohen E, Feinglass J, McGaghie W, Wayne D. Use of Simulation-Based Education to Reduce Catheter-Related Bloodstream Infections. Archives of Internal Medicine. 2009;169(15):1420.
29. Barsuk J, Ahya S, Cohen E, McGaghie W, Wayne D. Mastery Learning of Temporary Hemodialysis Catheter Insertion by Nephrology Fellows Using Simulation Technology and Deliberate Practice. American Journal of Kidney Diseases. 2009;54(1):70-76.
30. Barsuk J, McGaghie W, Cohen E, OʼLeary K, Wayne D. Simulation-based mastery learning reduces complications during central venous catheter insertion in a medical intensive care unit . Critical Care Medicine. 2009;37(10):2697-2701.
31. Kessler D, Auerbach M, Pusic M, Tunik M, Foltin J. A Randomized Trial of Simulation-Based Deliberate Practice for Infant Lumbar Puncture Skills. Simulation in Healthcare: The Journal of the Society for Simulation in Healthcare. 2011;6(4):197-203.
32. Wayne D, Didwania A, Feinglass J, Fudala M, Barsuk J, McGaghie W. Simulation-Based Education Improves Quality of Care During Cardiac Arrest Team Responses at an Academic Teaching Hospital. Chest. 2008;133(1):56-61.
33. Wayne D, Butter J, Siddall V, Fudala M, Linquist L, Feinglass J et al. Simulation-Based Training of Internal Medicine Residents in Advanced Cardiac Life Support Protocols: A Randomized Trial. Teaching and Learning in Medicine. 2005;17(3):202-208.
34. Wayne D, Fudala M, Butter J, Siddall V, Feinglass J, Wade L et al. Comparison of Two Standard-setting Methods for Advanced Cardiac Life Support Training. Academic Medicine. 2005;80(Supplement):S63-S66.
35. Wayne D, Butter J, Siddall V, Fudala M, Wade L, Feinglass J et al. Mastery learning of advanced cardiac life support skills by internal medicine residents using simulation technology and deliberate practice. Journal of General Internal Medicine. 2006;21(3):251-256.
36. Wayne D, Barsuk J, O'Leary K, Fudala M, McGaghie W. Mastery learning of thoracentesis skills by internal medicine residents using simulation technology and deliberate practice. Journal of Hospital Medicine. 2008;3(1):48-54.
37. McGaghie W, Issenberg S, Barsuk J, Wayne D. A critical review of simulation-based mastery learning with translational outcomes. Medical Education. 2014;48(4):375-385.
38. ten Cate O. Trust, competence, and the supervisor's role in postgraduate training. BMJ. 2006;333(7571):748-751.
39. ten Cate O, Billett S. Competency-based medical education: origins, perspectives and potentialities. Medical Education. 2014;48(3):325-332.
40. Yudkowsky R, Park Y, Downing S. Assessment in Health Professions Education. 1st ed. New York: Routledge; 2009.
